# Supplementary material for: Alternation in Peripheral B Cell Subpopulations Is a Potential Biomarker for Autoimmune Diseases—A Cross-Sectional Study
Source: Diagnostics (Basel). 2025 Jul 4;15(13):1710. doi: 10.3390/diagnostics15131710 (PMC12248792; doi:10.3390/diagnostics15131710)
Supplement: Supplementary file 1 [file diagnostics-15-01710-s001.zip › Table S1. Peripheral B cell subpopulation frequencies of males and females.pdf]

Table S1 (a). Peripheral B cell subpopulation frequencies of males and females in conrtol group

	**Male (*n* = 28)**	**Female (*n* = 37)**	***p*-Value**
WBCs (1000/μL)	6.5 (2.4)	6.5 (2.3)	0.884
Lymphocytes (% WBC)	31.1 (6.6)	28.9 (8.7)	0.408
B cells (% lymphocytes)	7.5 (4.9)	7.1 (5.1)	0.643
Naive (% B cells)	68.6 (21.8)	76.8 (16.5)	0.357
Naive Resting (% B cells)	41.8 (11.6)	43.0 (13.5)	0.726
IgM-negative Naïve (% B cells)	21.6 (17.0)	27.7 (24.5)	0.487
Memory (% B cells)	27.5 (18.6)	18.9 (15.3)	0.228
Non-switched (% B cells)	10.7 (9.5)	6.4 (7.2)	0.121
Switched (% B cells)	11.5 (8.5)	9.4 (8.5)	0.657
DN (% B cells)	1.5 (1.5)	1.6 (1.4)	0.415
CD38- DN (% B cells)	0.8 (1.2)	0.9 (0.8)	0.337
CD38+ DN (% B cells)	0.3 (0.6)	0.3 (0.4)	0.889
ASC (% B cells)	2.3 (1.8)	2.1 (1.9)	0.947
EPBs (% B cells)	1.3 (1.4)	1.4 (1.2)	0.801
PBs (% B cells)	0.3 (0.3)	0.4 (0.3)	0.119
Plasma cells (% B cells)	0.1 (0.1)	0.0 (0.1)	0.775

The continuous variables were analyzed using a Mann–Whitney U test and presented as median values (interquartile range). * $p < 0.05$. Abbreviations: WBCs, white blood cells; DN, double-negative B cells; ASCs, antibody-secreting cells; EPBs, early plasmablasts; PBs, plasmablasts.

Table S1 (b). Peripheral B cell subpopulation frequencies of males and females in patient group

	**Male (*n* = 25)**	**Female (*n* = 29)**	***p*-Value**
WBCs (1000/μL)	7.3 (2.7)	6.3 (3.7)	0.121
Lymphocytes (% WBC)	25.8 (11.8)	28.6 (20.5)	0.876
B cells (% lymphocytes)	8.7 (8.5)	7.1 (6.0)	0.362
Naive (% B cells)	63.0 (27.8)	69.0 (27.3)	0.456
Naive Resting (% B cells)	34.0 (20.0)	39.9 (13.2)	0.205
IgM-negative Naïve (% B cells)	19.8 (14.4)	17.5 (25.3)	0.249
Memory (% B cells)	24.4 (22.6)	15.1 (24.3)	0.775
Non-switched (% B cells)	5.8 (10.1)	3.2 (8.1)	0.087
Switched (% B cells)	11.2 (12.2)	9.0 (15.9)	0.979
DN (% B cells)	2.7 (6.8)	3.1 (3.9)	0.890
CD38- DN (% B cells)	1.5 (2.1)	1.5 (3.7)	0.677
CD38+ DN (% B cells)	0.6 (0.9)	0.5 (1.2)	0.742
ASC (% B cells)	4.3 (5.0)	2.8 (3.8)	0.187
EPBs (% B cells)	3.0 (3.6)	2.1 (3.7)	0.199
PBs (% B cells)	0.5 (0.6)	0.2 (0.5)	0.110
Plasma cells (% B cells)	0.0 (0.1)	0.0 (0.1)	0.923

The continuous variables were analyzed using a Mann–Whitney U test and presented as median values (interquartile range). * $p < 0.05$. Abbreviations: WBCs, white blood cells; DN, double-negative B cells; ASCs, antibody-secreting cells; EPBs, early plasmablasts; PBs, plasmablasts.
