# Supplementary material for: Alternation in Peripheral B Cell Subpopulations Is a Potential Biomarker for Autoimmune Diseases—A Cross-Sectional Study
Source: Diagnostics (Basel). 2025 Jul 4;15(13):1710. doi: 10.3390/diagnostics15131710 (PMC12248792; doi:10.3390/diagnostics15131710)
Supplement: Supplementary file 1 [file diagnostics-15-01710-s001.zip › Table S2. Peripheral B cell subpopulation frequencies of systemic lupus erythematosus patients with and without lupus nephritis .pdf]

Table S2. Peripheral B cell subpopulation frequencies of systemic lupus erythematosus patients with and without lupus nephritis

	**SLE Patients with lupus nephritis (*n* = 6)**	**SLE Patients without lupus nephritis (*n* = 7)**	***p*-Value**
WBCs (1000/μL)	6.2 (5.9)	5.8 (3.5)	0.534
Lymphocytes (% WBC)	24.4 (21.7)	17.6 (9.2)	0.836
B cells (% lymphocytes)	4.7 (7.4)	6.6 (17.6)	0.534
Naive (% B cells)	76.1 (46.0)	55.2 (15.3)	0.366
Naive Resting (% B cells)	61.6 (56.2)	38.3 (22.6)	0.366
IgM-negative Naïve (% B cells)	4.7 (19.1)	4.5 (8.8)	1.000
Memory (% B cells)	9.4 (22.4)	28.0 (41.8)	0.445
Non-switched (% B cells)	1.7 (1.3)	2.0 (7.8)	0.818
Switched (% B cells)	5.7 (19.4)	23.1 (30.0)	0.234
DN (% B cells)	6.1 (10.0)	6.6 (8.0)	0.945
CD38- DN (% B cells)	3.2 (7.2)	2 .8 (3.7)	0.731
CD38+ DN (% B cells)	1.5 (2.6)	1 .0 (2.1)	0.628
ASC (% B cells)	9.1 (14.6)	7 .8 (5.7)	0.731
EPBs (% B cells)	6.6 (13.0)	5.3 (4.3)	0.731
PBs (% B cells)	0.7 (2.0)	0.4 (0.6)	0.394
Plasma cells (% B cells)	0.0 (0.2)	0.0 (0.1)	0.857

The continuous variables were analyzed using a Mann–Whitney U test and presented as median values (interquartile range). * $p < 0.05$. Abbreviations: SLE, systemic lupus erythematosus; WBCs, white blood cells; DN, double-negative B cells; ASCs, antibody-secreting cells; EPBs, early plasmablasts; PBs, plasmablasts.
